# Knowledge, attitudes, and practice about protective ventilation among physical therapists

**DOI:** 10.1371/journal.pone.0331949

**Published:** 2025-09-19

**Authors:** Iara Sayuri Shimizu, Sângia Feucht Freire, Mayson Laercio de Araujo Sousa, Juliana Carvalho Ferreira

**Affiliations:** 1 Divisao de Pneumologia, Instituto do Coracao (InCor), Hospital das Clinicas HCFMUSP, Faculdade de Medicina, Universidade de Sao Paulo, Sao Paulo, SP, Brazil; 2 Coordenacao de Fisioterapia, Centro de Ciencias da Saude, Universidade Estadual do Piaui (UESPI), Teresina, PI, Brazil; 3 Department of Respiratory Therapy, College of Rehabilitation Sciences, Rady Faculty of Health Sciences, University of Manitoba, Winnipeg, Manitoba, Canada; Jashore University of Science and Technology (JUST), BANGLADESH

## Abstract

**Objectives:**

To measure knowledge, attitudes and practice (KAP) towards protective ventilation and identify factors associated with KAP among physical therapists in a country-wide survey.

**Methods:**

We conducted an online survey using a validated questionnaire with 55 items including individual and institutional information, KAP, and barriers to protective ventilation. The survey was distributed by email and social media. We calculated a total KAP score and scores for knowledge, attitudes, and practice, using a standardized scale from 0 to 100.

**Results:**

We included 408 participants from all states of Brazil. Median knowledge score was 80 (IQR 72–88) out of 100, with 95% respondents agreeing that they were familiar with the ventilatory settings to achieve protective ventilation, but 34% reported that airway pressures are not always discussed during rounds. Total KAP score had a median of 71 (62–79) out of 100. In the multivariate analysis, years of ICU experience, attending conferences, and ICU beds per physical therapist were independently associated with KAP score. The most significant barriers to protective ventilation were lack of education to provide low tidal volume ventilation and maintaining protective ventilation in pressure support. Participants reported there was an increase in the practice of protective ventilation during COVID-19 pandemic.

**Conclusions:**

In this countrywide study, physical therapists had good knowledge, attitudes, and practice regarding protective ventilation, and lack of education was an important factor associated with KAP. Discussing airway pressures during ICU rounds and developing specific training may improve awareness and practice of protective ventilation and impact patient outcomes.

## Introduction

Clinical trials have shown that protective ventilation based on low tidal volume and limited airway pressures reduces mortality in patients with Acute Respiratory Distress Syndrome (ARDS) [1,2] and prevents ventilator-induced lung injury [3,4]. More recently, studies have shown that protective ventilation may also benefit patients at risk of ARDS and COVID-19 [5,6]. Despite evidence of benefits, implementation of protective ventilation in clinical practice has been slow [7–9].

Barriers to the implementation of protective ventilation include lack of knowledge, concerns related to patient discomfort and need for sedation, impact on physiological parameters, and difficulty to measure patient’s height to calculate predicted body weight [10]. On the other hand, factors such as clinician experience, use of protocols and team training may be associated with increased adherence to protective ventilation [9,11–13].

Knowledge, attitudes, and practice (KAP) surveys can be used to identify barriers and facilitators to the adoption and implementation of evidence-based interventions. KAP surveys collect information on what is known, believed, and done in relation to a particular topic by a specific population, and identify knowledge gaps, behavioral patterns, and barriers to implementation [14]. A KAP survey focusing on protective ventilation has been used to describe the KAP among healthcare professionals in the United States and found that knowledge varied by caregiver type and experience [11]. However, limited data is available on KAP and barriers to the implementation of protective ventilation across multiple institutions or other settings.

Awareness of KAP and barriers to the use of protective ventilation is essential to improving its implementation and providing evidence-based care to patients [10,11]. Since patient care is a collaborative effort involving several health care professionals it is important to evaluate KAP among different health professionals [10,11].

Therefore, the aim of this study was to evaluate KAP and barriers to protective ventilation and identify individual and institutional factors associated with KAP among physical therapists in a country-wide survey.

## Materials and methods

### Study design and sample

This is a cross-sectional survey study. We included physical therapists who work in the intensive care unit (ICU) for the past six months. Exclusion criteria were refusal to participate and sign informed consent, physical therapists working in pediatric and neonatal ICU, and incomplete forms.

A combination of convenience and snowball sampling strategies were used to recruit participants. Initially, a convenience sample was obtained by distributing email invitations through professional organizations, including the Brazilian Association of Cardiorespiratory and Physiotherapy (ASSOBRAFIR), the Brazilian Intensive Care Medicine Association (AMIB), and regional physical therapy councils. Additionally, the survey was shared on social media platforms. Participants were encouraged to further disseminate the survey by sharing it with colleagues, professional societies, and institutions within their networks, thereby enabling snowball sampling. The recruitment period lasted from September 24 to December 31, 2021.

The study was approved by the Research Ethics Committee of the Heart Institute, University of São Paulo (approval number 3315210). Informed consent was obtained electronically from all participants.

The results were written according to the Strengthening the Reporting of Observational studies in Epidemiology (STROBE) reporting guideline [15].

### Survey design

Survey development was based on previously published surveys [10,11] to evaluate perceived knowledge, attitudes, and practice regarding protective ventilation among ICU physical therapists.

The initial draft had 24 demographic and professional items, 22 KAP items and four barriers items [11]. The survey was translated into Portuguese and validated in two steps. A panel of experts, consisting of seven physical therapists from different regions of Brazil, reviewed the survey for comprehensiveness, clarity, and relevance. After three rounds, two additional barriers and three questions about the impact of the COVID-19 pandemic were added. We piloted the final version of the survey on 33 physical therapists from diverse regions of the country.

The final survey was composed of 55 items, organized in three sections. The first section focused on demographic and professional information, including age, sex, time since completion of training, having a specialization in ICU or respiratory physical therapy, having an ICU specialist certification issued by the Federal Physical Therapy Council, experience in ICU, and participation in training and/or conferences. The second section included institutional information, such as hospital and ICU type, number of hospital and intensive care beds, and number of physical therapists in the ICU team. The third section measured KAP and barriers to protective ventilation.

KAP items were organized as five subjective knowledge items, four objective multiple-choice items, 10 items that assessed attitudes, three practice items evaluating behaviors, and six items on participant’s perception of barriers to protective ventilation.

We used a 6-point Likert scale, with the following response options: strongly agree, agree, neutral, disagree, strongly disagree, do not know. The statements alternated between positive and negative wording to avoid response set bias. Positively worded items were scored from 5 to 0 points, where 5 was attributed to strongly agree, and 1 was attributed to strongly disagree. We used reverse scores for negatively worded items, i.e., 5 was attributed to strongly disagree and 1 attributed to strongly agree. The “Do not know” option scored 0 points for all items.

Objective knowledge items were recoded as correct or incorrect (see supplementary material), and for each correct answer, five points were computed, assigning a final score from 0 to 20. This score was dichotomized into high knowledge (score > 10) and low knowledge (score ≤ 10), as previously defined [11].

We calculated separated scores for knowledge, attitudes, and practice, in additional to a total KAP score. Scores were calculated as the sum of individual items. The knowledge score was calculated by adding the number of points obtained in the subjective knowledge items and the points obtained in the objective knowledge items and ranged from 0 to 45 points. The attitude score ranged from 0 to 50 points, and the practice score ranged from 0 to 15 points. The total KAP score ranged from 0 to 110. We standardized scores by dividing the total number of points by the maximum number of points, times 100, resulting in a standardized score from 0 to 100. Therefore, the higher the score, the greater the knowledge, attitudes, and practice about protective ventilation. The barriers score ranged from 0 to 30, where the lower the score, the more important the barriers perceived to provide protective ventilation.

Data were collected and managed using REDCap electronic data capture tool [16].

### Statistical analysis

The sample size was calculated to perform a multiple regression model, considering an α of 0.05, a power of 0.8, and up to 9 independent variables, with a multiple correlation coefficient of 0.20, and found that the minimum required sample size was 367 patients (Stats to Do, China) [17].

Categorical variables were summarized as proportions. Continuous data variables were reported as mean and standard deviation or as medians and interquartile ranges as appropriate.

Linear regression models were used to examine the association of KAP score with individual factors (age, time since completion of training, years of ICU experience, ICU specialist board certification, ICU or respiratory specialization and participation in training and/or conferences) and institutional factors (availability of physical therapist in night shift, number of ICU beds per physical therapist, and institutional training offered by institution). Multicollinearity was calculated by the variance inflation factor (VIF) for each independent variable. Age, time since completion of training and years of ICU experience had a strong collinearity, and therefore we added years of ICU experience only to the multivariable model.

Multivariable linear regression model was built based on a conceptual causal diagram in the format of directed acyclic graph (DAG). Variables in the DAG conceptual model were selected based on prior knowledge and are depicted in the (S1 Fig).

We checked the internal consistency of the KAP score items with the Cronbach’s alpha coefficient, and we found α = 0.75 (IQR 0.7–0.77). Convergent validity was evaluated using a Spearman test between objective and subjective knowledge and found a weak positive correlation (rho = 0.34, p < 0.001).

All data were entered and analyzed using the software Statistical Package R, version 4.0.3. We considered p* *≤ 0.05 as statistically significant.

## Results

Five hundred and twenty-nine physical therapists accessed the survey. Of these, 121 were ineligible, therefore 408 participants were included in the study (S2 Fig).

The main characteristics of study respondents are presented in Table 1. Mean age was 33 ± 7 years old and 63% of respondents were female. Most respondents worked in public hospitals and had a median of 5 years of professional experience in the ICU.

**Table 1:** Characteristics of study respondents (n = 408).

Age, years – Mean (SD)	33 ± 7
**Sex – Female** – n (%)	255 (63%)
**Time since completion of training, years** – Median (IQR)	8 (3–13)
**ICU experience, years** – Median (IQR)	5 (2–10)
**ICU or Respiratory specialization** – n (%)	290 (71%)
**Roles in the ICU** – n (%)	
Coordinator	62 (15%)
Clinical	390 (97%)
Research	29 (7%)
Teaching	63 (16%)
**ICU specialist board certification** – n (%)	89 (22%)
**Participation in training and/or conferences** – n (%)	
None	28 (7%)
1–2 training/conferences in last 2 years	175 (43%)
3–5 training/conferences in last 2 years	140 (34%)
6 or more training/conferences in last 2 years	64 (16%)
**Physical therapists’ distribution per region in Brazil** – n (%)	
North	29 (7%)
Northeast	124 (31%)
Central west	20 (5%)
Southeast	178 (44%)
South	54 (13%)
**Hospital type** – n (%)	
Public	212 (52%)
Private	79 (20%)
Mixed	71 (17%)
Philanthropic	45 (11%)
**ICU type** – n (%)	
Medical	106 (26%)
Surgical	14 (4%)
Mixed	221 (54%)
Others	66 (16%)
**Hospital beds** – Median (IQR)	100 (17–250)
**ICU beds** – Median (IQR)	10 (10–19)
**Number of ICU beds per physical therapists** – Median (IQR)	8 (5–10)

Footnote: SD: Standard Deviation, IQR: Interquartile Range. Data are n. (%), unless otherwise stated: age was missing for 1 respondent, time since completion of training was missing for 1 respondent, ICU specialist certification was missing for 1 respondent, participation in training and/or conferences was missing for 1 respondent, hospital type was missing for 1 respondent, ICU type was missing for 1 respondent, and ICU beds was missing for 1 respondent. Sex was missing for 2 respondents, and ICU experience was missing for 2 respondents. Distribution per region was missing for 3 respondents, and hospital beds was missing for 3 respondents. Number of ICU beds per physical therapists was missing for 5 respondents. ICU or respiratory specialization was missing for 7 respondents, and main role in the ICU was missing for 7 respondents.

### Knowledge

**Fig 1:**
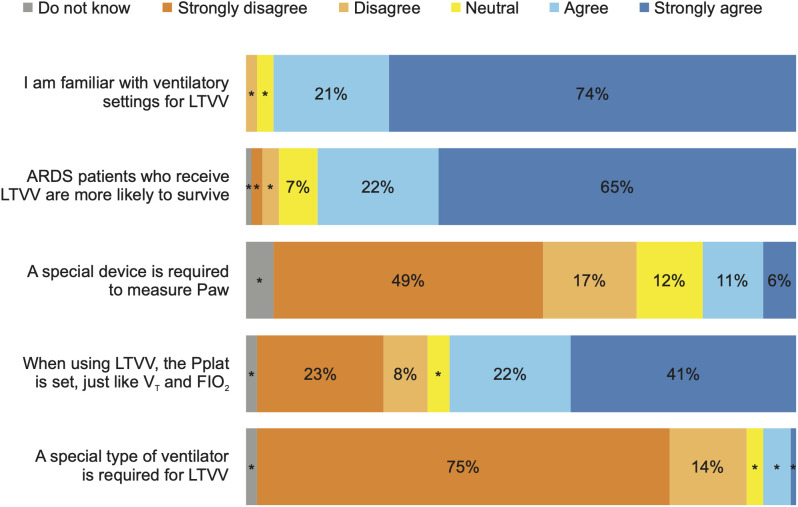
Knowledge about low tidal volume ventilation. Legend: LTVV: Low Tidal Volume Ventilation, ARDS: Acute Respiratory Distress Syndrome, Paw: Airway Pressure, Pplat: Plateau Pressure, VT: Tidal Volume, FiO2: Fraction of Inspired Oxygen. * ≤ 5%.

Fig 1 shows the performance on subjective knowledge about LTVV. Median knowledge score was 80 (IQR 72–88) out of 100. Most respondents (95%) agreed that they were familiar with the ventilatory settings to achieve LTVV. In contrast, 63% believed that it is necessary to set plateau pressure to provide LTVV.

Performance on the objective knowledge items was 10 (IQR 5–15) out of 20 points (S1 Table). The item with the highest percentage of correct responses was about keeping plateau pressure ≤30 cmH_2_O to offer protective ventilation.

Most participants, 248 (61%), had low knowledge in the objective items, and the median standardized knowledge, including subjective and objective items was 68 (IQR 53–80) of 100.

### Attitudes

**Fig 2:**
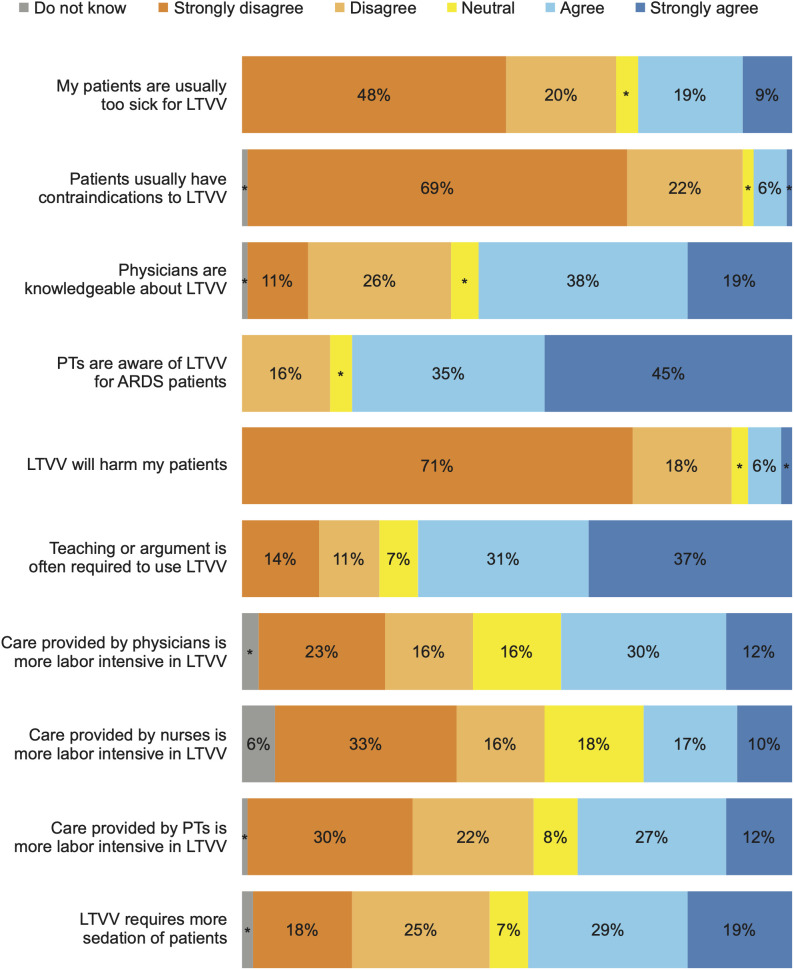
Attitudes toward low tidal volume ventilation. Legend: LTVV: Low Tidal Volume Ventilation, PT: Physical Therapist, ARDS: Acute Respiratory Distress Syndrome. * ≤ 5%.

The results of attitude’s items are shown in Fig 2. Median attitude score was 70 (IQR 60–80) out of 100. Attitudes regarding the need for more sedation to implement LTVV was diverging, but most respondents disagree that contraindications to use LTVV are common among ARDS patients.

### Practice

**Fig 3:**
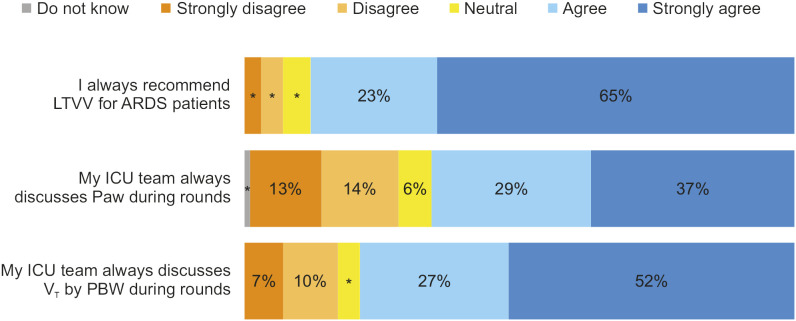
Practice regarding protective ventilation. Legend: LTVV: Low Tidal Volume Ventilation, ARDS: Acute Respiratory Distress Syndrome, ICU: Intensive Care Unit, Paw: Airway Pressure, VT: Tidal volume, PBW: Predicted Body Weight.

Fig 3 shows the practice regarding protective ventilation. Median practice score was 87 (IQR 67–100) out of 100. Most respondents said they use low tidal volume ventilation for ARDS except when there are contraindications, but airway pressures are not always discussed on rounds.

### Barriers

**Fig 4:**
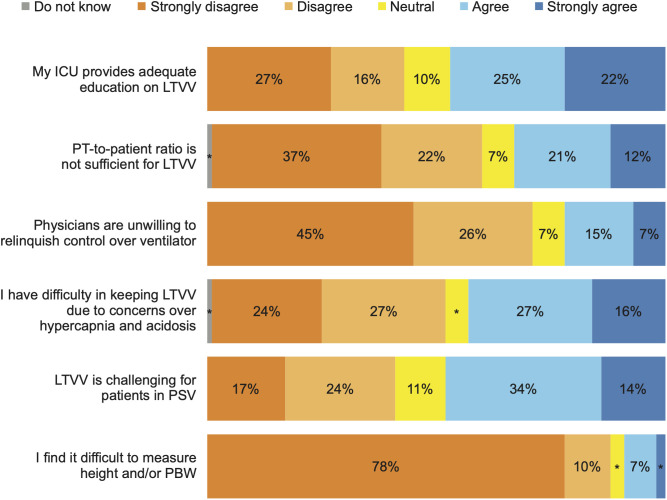
Barriers to protective ventilation. Legend: ICU: Intensive Care Unit, LTVV: Low Tidal Volume Ventilation, PT: Physical Therapist, PSV: Pressure Support Ventilation, PBW: Predicted Body Weight. * ≤ 5%.

The results of barriers are shown in Fig 4. Median barriers score was 70 (IQR 59–83) out of 100. The most significant barriers to the use of protective ventilation were lack of education to provide LTVV and maintaining protective ventilation for patients in PSV.

### Factors associated with KAP score

KAP score had a median of 71 (IQR 62–79) out of 100. In the multivariate analysis, years of ICU experience (ß 0.33, 95% CI 0.1–0.5, p* *= 0.001), attending conferences (ß 2.67, 95% CI 1.3–4.0, p < 0.001), and ICU beds per physical therapist (ß 0.40, 95% CI 0.1–0.7, p* *= 0.02) were independent predictors of higher KAP score (S2 Table).

### Impact of COVID-19 pandemic

Most physical therapists reported changes during the COVID-19 pandemic, with 362 (89%) agreeing that there was an increase in the practice of protective ventilation, 355 (87%) observed an increase in the number of ICU beds and physical therapists in the hospital, and 272 (67%) reported increased workload.

We found a significant association of increased practice of protective ventilation with KAP score (ß 6.3, 95% CI 2.6–10, p < 0.001) during the COVID-19 pandemic.

## Discussion

In this observational study including 408 Brazilian physical therapists from all regions of the country, we found that the participants had good knowledge, attitudes, and practice towards protective ventilation. Subjective knowledge scores were high, with 95% respondents reporting familiarity with the ventilatory settings to achieve LTVV, although performance on objective knowledge items was not as high. Discussion of airway pressures during rounds was reported as the least implemented practice related to protective ventilation, and the most significant barriers identified were lack of education and maintaining protective ventilation for patients in PSV. In a multivariable analysis, we found that factors such as more years of ICU experience, attending conferences and number of beds per physical therapist were associated with higher KAP score. Additionally, most participants reported an increase in the practice of protective ventilation during the COVID-19 pandemic, associated with increased workload.

This is the first study to describe KAP towards protective ventilation among physical therapists in a low and middle-income country (LMIC). Previous studies were conducted in high income countries where ICU human resources are very different, not only in terms of number of health professionals per ICU bed, but also in terms of training and roles [8,11]. In Brazil and other countries, physical therapists manage the mechanical ventilator [18,19], while in the USA and Canada, respiratory therapists (RTs) are responsible for the adjustment of the ventilator [20]. More importantly, these previous studies focused on other health professionals, with little or no representation of physical therapists or respiratory therapists [11,21,22] and primarily identified barriers, rather than KAP [8–10,23].

Participants showed relatively good knowledge about LTVV, with a median score of 10 out 20 points, unlike previous studies that observed lower knowledge (e.g., mean of 1.1 out of 4 among nurses) [11,22]. Previous studies are 15 years old, so the knowledge gap may have decreased after new guidelines [24,25]. It is also important to note that those studies used different assessment tools and involved different populations, which limits direct comparisons. Most participants reported that they were familiar with the ventilatory settings of LTVV and believed that LTVV improves survival for ARDS patients, but more than half believed that plateau pressure was set. One interpretation of this apparent inconsistency is that participants in fact lack the knowledge they claimed to have about the ventilatory settings required for LTVV.

Only a third of participants had high knowledge in the objective items. The objective item with best performance was about keeping the plateau pressure <30 cmH_2_O. Limiting plateau pressure has been a widely disseminated recommendation for several years [2,24,25], and good knowledge about it has been reported [11,21]. The difference in performance between subjective and objective items was also observed in another study that used the same knowledge items [22]. The contrast may be due to several factors, including differences between perception of knowledge and actual knowledge. This finding highlights the importance of including objective items in KAP surveys to assess the ability to solve more complex problems [26].

Participants had generally positive attitudes towards protective ventilation, with median attitude score of 70 out of 100. This contrast with previous studies that reported mean attitude score ranging from 27 to 40 out of 100 [10,11]. Moreover, most respondents disagreed with the statement that ARDS patients usually have contraindications to use LTVV, and with the statement that LTVV harms the ARDS patients. This be due to the dissemination of the benefits of protective ventilation, for patients with or without ARDS [2,4,27,28].

There was divergence of attitude when the participants were asked if protective ventilation requires more sedation of patients compared with conventional ventilation. This may reflect a perception that severely ill patients need to be deeply sedated [29,30], and that protective ventilation requires increased sedation for effective implementation [10,11], contrary to recent evidence that deep sedation does not improve outcomes in ARDS [31].

Participants reported good practices regarding protective ventilation. Most participants reported that they always recommend protective ventilation for ARDS patients. However, that may not correspond to actual practices implemented at the bedside, since studies show that professionals’ perception may be different from their behavior [8,9] and that adherence to protective ventilation for ARDS patients is low [32,33]. Participants reported that airway pressure and V_T_ by PBW are not always discussed during rounds, underscoring a lack of strategies to incorporate protective ventilation discussions into routine in ICUs [27,34] The use of structured checklists incorporating protective ventilation strategies during multidisciplinary rounds [9,13,35].

Our study found varying perceptions about barriers to initiating and keeping LTVV. Half of the participants reported having difficulty keeping LTVV due to concerns over hypercapnia and acidosis, despite evidence that permissive hypercapnia associated with LTVV does not increase mortality [2,36]. There were also mixed results on the difficulty of keeping low tidal volume in PSV, reflecting uncertainty around the balance of potential risks and benefits of spontaneous breathing in ARDS [37,38].

Another identified barrier was access to continued education, participants reported lack of training and evidence shows that training improves adherence to LTVV [12,39] and may improve outcomes [40]. This data reinforces that there is a need to develop educational strategies regarding mechanical ventilation and protective ventilation and of studies that estimate the impact of training on adherence to best practices, mitigation of healthcare worker burnout and patient outcomes.

We did not observe the physician’s unwilling to relinquish control of the ventilator as an important barrier, differing from previous studies [10,11], possibly because in Brazil, many physical therapists play an active role in the adjustment of mechanical ventilation [18,41].

We found that attending conferences were associated with higher KAP score. Interestingly, the association between higher KAP score and participation in conferences is in line with the participants’ perception that lack of training in protective ventilation was a barrier to its implementation and reinforces the importance of educational interventions involving a multidisciplinary team, paired with treatment protocols, to improve outcomes [7,12] We also found that number of years of ICU experience was associated with higher KAP, as previously shown [10,11].

The ratio of ICU beds to physical therapist was the only institutional factor associated with higher KAP score. This association can be influenced by several factors, including, ICU complexity, case mix, and the fact that academic ICUs follow the federal regulation of one physical therapist per ten ICU beds.

Participants reported that there was an increase in the practice of protective ventilation during COVID-19 pandemic, and we found a positive association of this increased practice with KAP score. Protective ventilation has been recommended for critically ill patients with COVID-19 [42,43] and associated with ICU survival [5,6].

## Limitations

Our study has several limitations. First, we were unable to estimate the response rate, given that the survey link was sent by email or social media. To mitigate this limitation, we partnered with country-wide professional associations, and obtained a significant number of responses from all estates of the country. Second, KAP surveys do not capture the full spectrum of knowledge, attitudes, and practice at the bedside. However, they can provide valuable information from the participants’ perspective. We used a validated survey that was published in 2007 and may be outdated in some respects. Third, our data was collected during the COVID-19 pandemic, which was marked by greater discussion about protective ventilation, and it is possible that KAP were overestimated.

## Strengths

Our study has several strengths. It included a large, nationwide sample of physical therapists from all regions of Brazil, enhancing the representativeness and generalizability of our findings. The recruitment strategy involved partnerships with professional associations, which helped ensure broad reach and engagement across diverse settings. The survey combined both subjective and objective items, providing a nuanced assessment of perceived versus actual knowledge. We also conducted multivariable analyses to identify independent factors associated with higher KAP scores, which may inform targeted interventions. Although data were collected during the COVID-19 pandemic, as acknowledged in our limitations, this timing also provided valuable insight into shifts in clinical practice patterns.

## Conclusions

In this countrywide KAP study, we found that ICU physical therapists of a large LMIC had good knowledge, attitudes, and practice toward protective ventilation, and identified barriers and facilitators. We also found that the COVID-19 pandemic increased the practice of protective ventilation. These results suggest that including systematic discussions about tidal volume and plateau pressure during ICU rounds and developing specific training may improve awareness and practice of protective ventilation, which in turn, could improve patient outcomes.

## Supporting vinformation

S1 Fig Causal diagram in the format of directed acyclic graph (DAG) showing the conceptual model of association between KAP and other relevant covariates. Footnote: ICU: Intensive Care Unit. PT: Physical therapist. This conceptual model using Directed acyclic graph (DAG) shows relevant variables associated with KAP. Arrows indicate a suspected direct causal effect of one variable on another variable. Individual and institutional factors are the predictor, shown in green. A multivariable analysis of the effect of individual and institutional factors on KAP show.
(DOCX)

S2 Fig Study flowchart. (DOCX)

S1 Table Performance on Knowledge Test. (DOCX)

S2 Table Individual and institutional factors associated with KAP score about protective ventilation. (DOCX)
