# Supplementary material for: Knowledge, attitudes, and practice about protective ventilation among physical therapists
Source: PLoS One. 2025 Sep 19;20(9):e0331949. doi: 10.1371/journal.pone.0331949 (PMC12448968; doi:10.1371/journal.pone.0331949)
Supplement: S1 Fig — Footnote: ICU: Intensive Care Unit. PT: Physical therapist. This conceptual model using Directed acyclic graph (DAG) shows relevant variables associated with KAP. Arrows indicate a suspected direct causal effect of one variable on another variable. Individual and institutional factors are the predictor, shown in green. A multivariable analysis of the effect of individual and institutional factors on KAP show. (DOCX) [file pone.0331949.s001.docx]

**
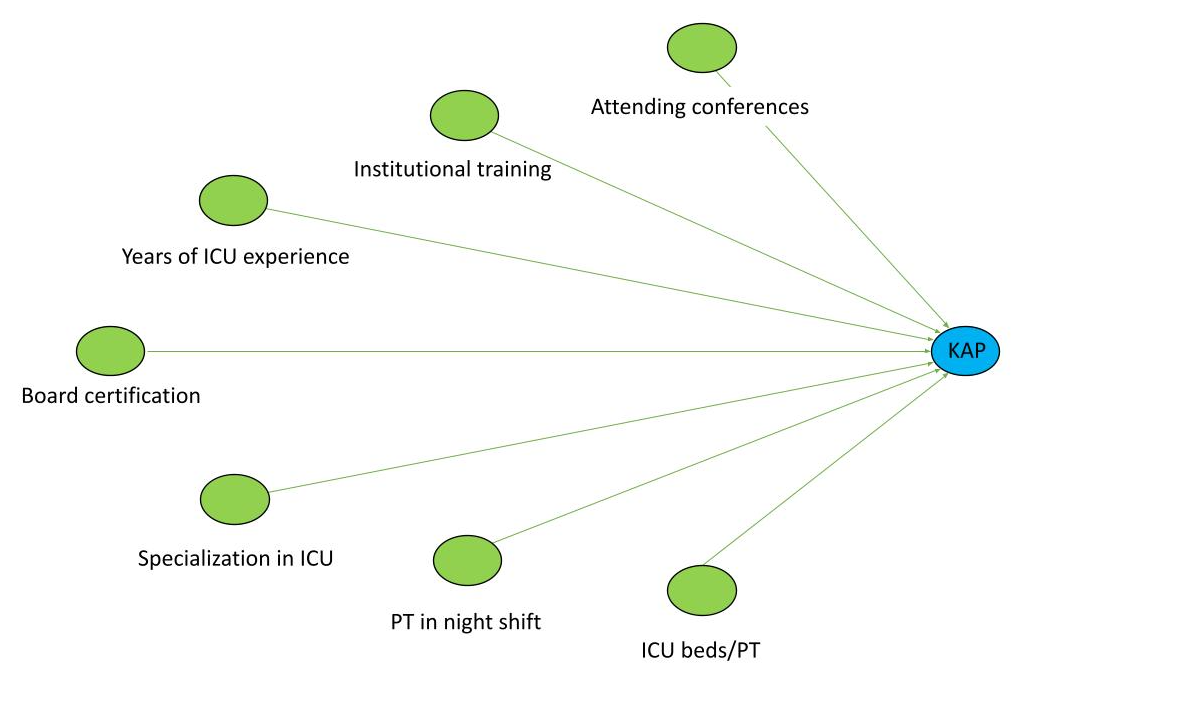
**

**S1 Fig. Causal diagram in the format of directed acyclic graph (DAG) showing the conceptual model of association between KAP and other relevant covariates.**

Footnote: ICU: Intensive Care Unit. PT: Physical therapist. This conceptual model using Directed acyclic graph (DAG) shows relevant variables associated with KAP. Arrows indicate a suspected direct causal effect of one variable on another variable. Individual and institutional factors are the predictor, shown in green. A multivariable analysis of the effect of individual and institutional factors on KAP show.
