# Supplementary material for: Knowledge, attitudes, and practice about protective ventilation among physical therapists
Source: PLoS One. 2025 Sep 19;20(9):e0331949. doi: 10.1371/journal.pone.0331949 (PMC12448968; doi:10.1371/journal.pone.0331949)
Supplement: S2 Fig — (DOCX) [file pone.0331949.s002.docx]

Assessed for eligibility (n=529)

Excluded (n=121)

- Declined to participate (n=6)
- Does not work in ICU (n=84)
- Pediatric and neonatal ICU (n=20)
- Incomplete forms (n=11)

Included in the analysis (n=408)

**S2 Fig. Study flowchart**
