# Supplementary material for: Knowledge, attitudes, and practice about protective ventilation among physical therapists
Source: PLoS One. 2025 Sep 19;20(9):e0331949. doi: 10.1371/journal.pone.0331949 (PMC12448968; doi:10.1371/journal.pone.0331949)
Supplement: S1 Table — (DOCX) [file pone.0331949.s003.docx]

**S1 Table. Performance on Knowledge Test**

| **Item** | **Correct response** |
| --- | --- |
| **An important goal of lung protective ventilation for ARDS is to:**  1. Maintain PaO_2_ >60 mm Hg at the lowest possible PEEP  2. Keep peak airway pressure <35 cm H_2_O  **3. Keep plateau pressure <30 cmH_2_O***  4. Keep tidal volume <10 mL/kg of predicted body weight  5. I don’t know | 287 (70%) |
| **An ARDS patient is receiving protective ventilation with a V_T_ of 6 mL/kg PBW, respiratory rate of 35 breaths/min, P_plat_ of 26 cmH_2_O. What would be your next intervention if the reported ABG demonstrates a pH of 7.25 and a PaCO_2_ of 55 mmHg?**  1. Increase the respiratory rate to 38 breaths/min  2. Increase the tidal volume to 7 mL/kg  **3. Initiate bicarbonate infusion***  **4. Repeat ABG in 4 hours***  5. I don’t know | 231 (57%) |
| **In an ARDS patient receiving a V_T_ of 5 mL/kg PBW, in which circumstance below would you increase the tidal volume by 1 mL/kg?**  1. Peak pressure is <30 cmH_2_O  2. Arterial pH 7.25 and respiratory rate of 25 breaths/min  **3. Plateau pressure is ≤25 cmH_2_O***  4. PaO_2_ is 55 mm Hg on FiO_2_ of 0.5 and PEEP of 10 cmH_2_O  5. I don’t know | 154 (38%) |
| **An ARDS patient is receiving a V_T_ of 6 mL/kg PBW, respiratory rate of 30 breaths/min, FiO_2_ of 0.8, and PEEP of 14 cmH_2_O. The peak pressure is 38 cmH_2_O, and P_plat_ is 28 cmH_2_O. What would it be the next intervention if the reported ABG on this ventilatory setting is: pH= 7.31, paCO_2_= 58 mmHg, and PaO_2_= 59 mmHg?**  1. Increase FiO_2_ to 0.9  2. Increase plateau pressure by 2 cmH_2_O  3. Decrease tidal volume by 1 mL/kg IBW  **4. No changes on current ventilatory settings***  5. I don’t know | 172 (42%) |

**Footnote:** * indicates correct response(s)

ARDS: Acute Respiratory Distress Syndrome, PaO_2_: Partial pressure of oxygen, PEEP: Positive Expiratory End Pressure, V_T_: Tidal Volume, PBW: Predicted Body Weight, P_plat_: Plateau Pressure, ABG: Arterial Blood Gas, PaCO_2_: Partial pressure of carbon dioxide, FiO_2_: Fraction of Inspired Oxygen.
