# Supplementary material for: Knowledge, attitudes, and practice about protective ventilation among physical therapists
Source: PLoS One. 2025 Sep 19;20(9):e0331949. doi: 10.1371/journal.pone.0331949 (PMC12448968; doi:10.1371/journal.pone.0331949)
Supplement: S2 Table — (DOCX) [file pone.0331949.s004.docx]

**S2 Table. Individual and institutional factors associated with KAP score about protective ventilation**

| **Factors** | **Beta value**  **univariate** | ***P*** | **Beta value Multivariate** | ***P*** |
| --- | --- | --- | --- | --- |
| **Attending conferences** | 3.10 (1.7 – 4.5) | <0.001 | 2.67 (1.3 – 4.0) | <0.001 |
| **Institutional training** | 3.27 (0.9 – 5.6) | <0.01 | 2.10 (-0.1 – 4.3) | 0.06 |
| **Years of ICU experience** | 0.43 (0.3 – 0.6) | <0.001 | 0.33 (0.1 – 0.5) | 0.001 |
| **Board certification** | 5.83 (3,0 – 8.6) | <0.001 | 2.09 (-0.9 – 5.1) | 0.17 |
| **Specialization in ICU** | 2.52 (-0,1 – 5.1) | 0.06 | 1.99 (-0,4 – 4.4) | 0.11 |
| **Physical therapist in night shift** | 2.47 (-3.8 – 8.7) | 0.44 | 0.74 (-5.0 – 6.5) | 0.80 |
| **ICU beds/physical therapist** | 0.33 (0 – 0.7) | 0.07 | 0.40 (0.1 – 0.7) | 0.02 |

ICU: Intensive Care Unit. *P* values obtained with linear regression, univariate and multivariate.
